# TRIM22. A Multitasking Antiviral Factor

**DOI:** 10.3390/cells10081864

**Published:** 2021-07-23

**Authors:** Isabel Pagani, Guido Poli, Elisa Vicenzi

**Affiliations:** 1 Viral Pathogenesis and Biosafety Unit, IRCCS-Ospedale San Raffaele, 20132 Milan, Italy; pagani.isabel@hsr.it; 2 Human Immuno-Virology Unit, IRCCS-Ospedale San Raffaele, 20132 Milan, Italy; poli.guido@hsr.it; 3 School of Medicine, Vita-Salute San Raffaele University, 20132 Milan, Italy

**Keywords:** TRIM22, DNA and RNA viruses, HIV-1, influenza A virus, interferons

## Abstract

Viral invasion of target cells triggers an immediate intracellular host defense system aimed at preventing further propagation of the virus. Viral genomes or early products of viral replication are sensed by a number of pattern recognition receptors, leading to the synthesis and production of type I interferons (IFNs) that, in turn, activate a cascade of IFN-stimulated genes (ISGs) with antiviral functions. Among these, several members of the tripartite motif (TRIM) family are antiviral executors. This article will focus, in particular, on TRIM22 as an example of a multitarget antiviral member of the TRIM family. The antiviral activities of TRIM22 against different DNA and RNA viruses, particularly human immunodeficiency virus type 1 (HIV-1) and influenza A virus (IAV), will be discussed. TRIM22 restriction of virus replication can involve either direct interaction of TRIM22 E3 ubiquitin ligase activity with viral proteins, or indirect protein–protein interactions resulting in control of viral gene transcription, but also epigenetic effects exerted at the chromatin level.

## 1. Introduction

Innate immunity represents the frontline defense against viruses, aiming at preserving the host from viral invasion. Part of this complex network of cells and soluble factors is the intrinsic capacity of every cell to trigger a set of intracellular responses to viral infection in order to curtail its replicative capacity and further viral spreading. In fact, the cell response to viral entry is rapid and unspecific as both viral RNA and DNA genomes are sensed rapidly after their release in the cytoplasm by exposing evolutionarily conserved pathogen-associated molecular patterns (PAMP) to cellular germline-encoded pattern recognition receptors (PRRs) [1]. Viral component recognition initiates a signaling cascade that ultimately leads to transcription of pro-inflammatory cytokines and of type I interferons (IFNs), namely, IFN-β, firstly, and then IFN-α, the latter actually being a mixture of several proteins [2]. Type I IFNs bind to the IFN-α receptor (IFNAR) [3] in order to induce the expression of hundreds of IFN-stimulated genes (ISGs) that interfere with distinct stages of virus replication [4,5]. Among these ISGs, many TRIM proteins have been described to exert antiviral functions [5,6,7].

More than 80 TRIM proteins have been identified to share common structural features. TRIM proteins are characterized by an RBCC motif composed of an *N*-terminus domain, followed by a central region with one or two B boxes and a coiled-coil (CC) region. The RBCC motif is flanked by a *C*-terminus domain [8]. The *N*-terminus domain, defined as a RING (Really Interesting New Gene), is endowed with E3 ubiquitin ligase activity [9]. The CC domain is characterized by structural features that favor protein–protein interactions with different TRIM family members [10,11], but also other proteins [12]. The *C*-terminus domain is the most variable region among the TRIM proteins, and it is used to classify them into families [13,14].

TRIM protein members are classified into 11 families (from C-I to C-XI) based on their overall domain structure, with one group of TRIM proteins remaining unclassified due to a lack of a RING domain (e.g., TRIM14 and TRIM20) [13,15]. Many TRIM proteins have an antiviral function, and most of them belong to the C-IV family that represents the largest family with 34 members. This family is characterized by having a SPRY domain, or a SPRY region, in combination with a PRY domain to form a B30.2 domain at the *C*-terminus following the CC region. The B30.2 domain was originally identified as a protein domain encoded by a single exon (called B30-2) in the human major histocompatibility complex class I (MHC-I) region [16], and in genes involved in autoimmune and genetic diseases [17]. The SPRY domain was identified as a conserved domain in the non-receptor tyrosine kinase spore lysis A (splA) of *Dictyostelium discoideum*, and in mammalian ryanodine receptors (RyR) [18]. TRIM22 is characterized by a B30.2 domain including PRY and SPRY regions [19].

The member of the TRIM family that has historically received more attention as antiviral factor, particularly as an anti-HIV-1 determinant, is TRIM5α [20]. Interestingly, the *TRIM5* gene is located on chromosome 11 adjacent to the *TRIM22* gene [21]. Their proximity has been linked to a dynamic history of gene expansion and loss in mammals. For example, the cow genome encodes TRIM5 but has lost TRIM22, and vice versa, the dog genome encodes TRIM22 but has lost TRIM5. In primates, TRIM22 is present, although signatures of positive selection have been detected in the CC and B30.2 domains, suggesting a long history of interactions with viral pathogens but also endogenous retroviruses [21].

Among the several members of the TRIM family with antiviral activity, we have focused this article on TRIM22 as it targets multiple viruses by exploiting different mechanisms of inhibition. As the TRIM22 *N*-terminus domain is endowed with E3 ubiquitin ligase activity, poly-ubiquitination of viral proteins leads to their proteasome degradation, whereas the CC domain is engaged in more complex protein–protein interactions with less defined mechanism(s) of viral restriction [22,23].

## 2. TRIM22 Expression and Protein Localization

TRIM22, also known as Stimulated Trans-Acting Factor of 50 kDa (Staf50), was first discovered in a cDNA library screening of IFN-α/β-treated Daudi B cells as a gene that was transcriptionally upregulated [24]. TRIM22 is expressed in peripheral blood lymphocytes (PBMC) in response to IFN-α stimulation [24] and constitutively expressed in several human tissues, where it is highly upregulated in response to both type I and type II IFNs [25]. Indeed, the expression of many other TRIM family members is induced by type I and type II IFNs in PBMC [26], suggesting that TRIM proteins represent important mediators of the antiviral response. The 5′ flanking region of the *TRIM22* gene contains two regions matching the consensus sequence for an IFN-stimulating response element (ISRE), which are capable of binding IFN regulatory factor 1 (IRF-1) and are important for sensing the stimulation by type I and II IFNs, as well as for basal TRIM22 expression [27]. In addition to IFNs, TRIM22 expression is also modulated in response to several viruses and viral antigens [25]. For example, it is upregulated after infection of rubella virus and Epstein–Barr virus (EBV), but it is downregulated during infection with human papillomavirus type 31 [28], or by the hepatitis B virus (HBV) X protein, thereby allowing HBV to evade the host immune response [29].

The antiviral functions of TRIM22 are also dependent on its subcellular localization as it has been reported to be present both in the cytoplasm [10,30] and in the nucleus [31,32]. This distinct localization has mostly been studied in in vitro systems of ectopic expression. TRIM22 coupled to the green fluorescence protein (GFP) was localized in cytoplasmic bodies in U2OS cells [10], whereas another study reported a diffuse accumulation of the TRIM22-GFP fusion protein surrounding the nucleus of COS-7 cells [30]. A similar localization was observed in HeLa cells expressing endogenous TRIM22 [30]. In contrast, a c-myc-tagged TRIM22 expressed in human PBMC was localized exclusively in the nucleus [33]. Nevertheless, the nuclear expression of TRIM22 is dependent on the B30.2 domain [34], although it has been reported that both a deletion mutant of the RING domain and a cysteine mutant in position 15 of the RING domain disrupt its nuclear localization in HepG2 cells [31]. Furthermore, endogenous expression of TRIM22 has been selectively reported in the nucleus of HeLa cells and U937 cells [35]. The nuclear expression is characterized by the formation of nuclear bodies (NB) similar to TRIM19/PML NB, another member of the C-IV family with antiviral functions [36]. Indeed, TRIM19/PML NB are complex aggregates of proteins that not only include TRIM22 but also the transcription factors class II transactivator (CTIIA) and specificity protein-1 (Sp1), as well as Cyclin T1 (CyT1) [37]. These NB favor chromatinization and silencing of viral genomes [38], as in the case of HIV-1 that persists in latently infected cells [39,40].

In the next paragraphs, we will discuss the role of TRIM22 as an antiviral protein against specific viruses.

## 3. HIV-1

### 3.1. Life Cycle

**Figure 1:**
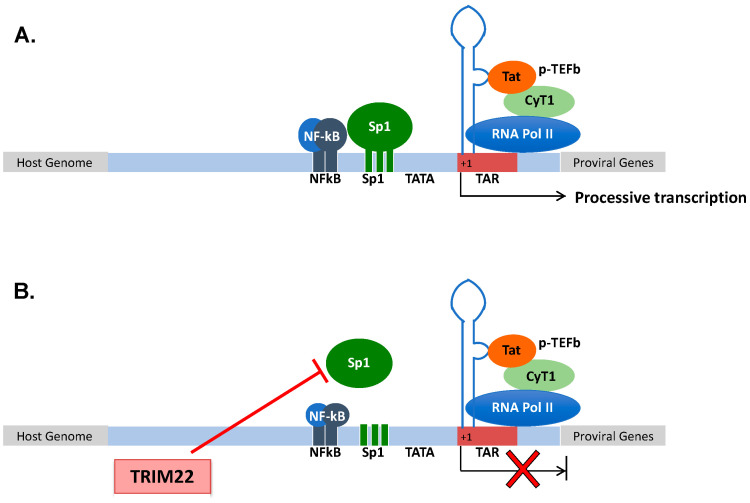
(**A**) Structural organization of the HIV-1 promoter. HIV-1 transcription starts at the promoter region in the 5′ LTR. Processive HIV-1 transcription is driven by the Tat protein that recruits the p-TEFb complex to the TAR RNA. pTEFb promotes the phosphorylation of the RNA Pol II, enabling the elongation of the viral transcripts. Upstream of the initiation of transcription (+1), three Sp1 and two NF-kB binding sites control the levels of basal transcription and response to inflammatory signals, respectively. (**B**) TRIM22 inhibits HIV-1 basal transcription by preventing Sp1 binding to the HIV-1 promoter, thus contributing to the maintenance of latent HIV-1 infection.

Human immunodeficiency virus type 1 (HIV-1) is a member of the lentivirus genus of the *retroviridae* family that causes a lethal condition known as AIDS (acquired immunodeficiency syndrome) in humans by infecting CD4^+^ T lymphocytes, causing their depletion and profound immunodeficiency, leading to opportunistic infections and cancer [41]; in addition to CD4^+^ T lymphocytes, HIV-1 also infects mononuclear phagocytes that are not depleted. After infection, the viral RNA genome is retrotranscribed into DNA that is then integrated as proviral DNA in the host genome [42]. The provirus is actively transcribed during a productive infection by the combined action of the viral protein Tat and of the cellular transcription machinery [43]. Tat is a virus-encoded transcriptional transactivator that binds to the RNA secondary structure of the transactivation region (TAR) of the 5′ long terminal repeat (LTR) (+1 to +59) [44,45] (Figure 1A). Once Tat is bound to the TAR RNA, it recruits a protein complex named positive transcription elongation factor b (p-TEFb) aimed to elongate the viral transcripts. p-TEFb is formed by the regulatory subunit CyT1 and the kinase subunit cyclin-dependent kinase 9 (CDK9) that phosphorylate the RNA polymerase II (Pol II) to increase its processivity. However, Tat elongation activity requires a basal transcription that is under the control of the upstream regulatory sequences, namely, three Sp1 and two nuclear factor kappa-light-chain-enhancer of activated B cells (NF-kB) binding sites that respond to pro-inflammatory signals [46]. The lack of NF-kB and Sp1 binding to the promoter, or the lack of recruitment of negative transcription factors to their DNA binding sites maintains a state of proviral latency [47]. In this regard, latently infected cells (mostly CD4^+^ T cells) are considered the main obstacle to virus eradication in that they are not affected by combination antiretroviral therapy (cART) [48,49]. The accomplishment of a full HIV-1 life cycle is essential for viral spreading, and it is counteracted by numerous host determinants collectively defined as restriction factors that are constitutively expressed prior to infection and/or are rapidly induced upon pathogen exposure [50]. Among these, other members of the TRIM family have been shown to play a significant role in preventing or containing HIV-1 replication, including TRIM5α [51], TRIM11 [52], TRIM28 [53], TRIM33 [54], TRIM34 [55] and TRIM37 [56].

### 3.2. TRIM22 Restriction of HIV-1

Since its discovery in 1995, TRIM22 has been characterized for its capacity to impair HIV-1 transcription [24]. Then, TRIM22 was demonstrated to inhibit HIV-1 replication in promonocytic cell lines, and in primary human monocyte-derived macrophages (MDM) [35,57]. Of interest, TRIM22 was shown to inhibit the basal activity of the HIV-1 promoter while not interfering with either the Tat-dependent or NF-kB-mediated upregulation of viral transcription, although it inhibited HIV-1 LTR-mediated gene expression induced by phorbol esters and ionomycin [35]. More recently, TRIM22 was shown to specifically interfere with Sp1-dependent transcription (Figure 1B). Sp1 is a zinc finger transcription factor constitutively expressed in many cell types that binds to GC-rich motifs present in many promoters, and it is involved in many cellular processes, including cell differentiation [58,59], cell growth [60], apoptosis [61], DNA damage response and chromatin remodeling [62].

Although TRIM22 (as with all the other TRIM proteins) does not bind directly to DNA sequences, it prevented the binding of Sp1 to its consensus sites in the HIV-1 LTR, as demonstrated by chromatin immunoprecipitation analysis [63] (Figure 1B).

Recent studies have demonstrated that TRIM22 plays a role in the maintenance of HIV latency in a Tat-independent context, highlighting the effect of TRIM22 on the LTR promoter region, and suggesting a contribution of this protein to the epigenetic silencing of the provirus [64]. More recently, another ISG, namely, interferon-γ inducible protein 16 (IFI16), was shown to restrict HIV-1 by sequestering the transcription factor Sp1, thereby inhibiting viral gene expression [65].

While TRIM22 E3 ubiquitin ligase activity was shown to be required for its interference with the release of HIV-1 particles, likely by interfering with post-translational modifications of HIV-1 Gag proteins [66], it was not required for TRIM22 inhibition of HIV-1 transcription. Therefore, the precise mechanism of TRIM22 interference with HIV-1 transcription is still partially unidentified [35]. TRIM22 did not cause the downregulation of Sp1 expression; nonetheless, TRIM22 inhibited the binding of Sp1 to the HIV-1 promoter, suggesting that a protein complex formed by TRIM22 and other cellular proteins could sequester Sp1, an interpretation supported by the observation that the CC domain of TRIM proteins mediates protein–protein interactions [11]. In this regard, it is worth noting the identification of two single-nucleotide missense polymorphisms (SNP) in the CC domain associated with a loss of inhibition of HIV-1 transcription and HIV-1 disease severity [67]. These two SNPs were discovered by comparing a TRIM22 sequence (as published in GenBank: NM_006074.4) with that of cell clones of the human promocytic cell line U937 that are either non-permissive (“Minus clones”) or permissive (“Plus clones”) to HIV-1 replication [68]. The two SNPs cause an A-to-G transition of SNP rs7935564 with an asparagine-to-aspartic acid substitution in position 155 (Asn155Asp, SNP1), whereas a C-to-G transversion of SNP rs1063303 causes a substitution of a threonine with arginine in position 242 (Thr242Arg, SNP2). Indeed, these two missense mutations affected HIV-1 replication in vitro as PBMC from individuals with the Asn155 and Thr242 haplotypes replicated HIV-1 less efficiently than PBMC with the other mutations. These results were consistent with the ability of TRIM22 to inhibit HIV-1 transcription in vitro. Conversely, the SNP1G variant alone was significantly more frequent in a cohort of HIV-1-infected individuals with advanced disease in comparison to long-term non-progressors (LTNP) or normal progressors [67].

Overall, these results unveiled a role of TRIM22 as a silencer of basal HIV-1 transcription, favoring the maintenance of a state of proviral latency.

## 4. Influenza A Virus (IAV)

### 4.1. IAV Infection

Influenza viruses are single-stranded, negative-sense, enveloped RNA viruses of the *orthomixoviridae* family with a segmented genome composed of eight independent RNA fragments, each one encoding for structural and non-structural proteins. According to the antigenic differences between the nucleoprotein (NP) and matrix (M) protein, influenza viruses can be classified into three types, namely, A, B and C. Although all three types of influenza viruses can naturally infect humans, only the type A virus has a wide range of animal host species, including birds, swine, horses and other mammals [69], whereas the identification of influenza B and C viruses in animal hosts is sporadic [70,71].

IAVs have been extensively studied due to their ability to cause highly contagious diseases in humans and animals (such as poultry, swine and horses), with potentially fatal outcomes [69]. Their intrinsic nature is to continuously change the antigenicity by accumulating point mutations on the surface glycoproteins to escape the existing immunity established by previous infection or vaccination (so-called “antigenic drift”) [69,72]. Furthermore, they cause pandemics by the so-called “antigenic shift”, during which new antigenic subtypes are introduced, by segment reassortment, into an immunologically naïve host population. Further adaptations occur to facilitate transmission in the new host species [73]. Although many global pandemics and major epidemics have occurred at regular intervals during human history [74], during the last century, however, four pandemics have been documented in 1918, 1957, 1968 and 2009 [75]. Then, due to the replicating nature of influenza viruses and the pressure of the immune response, the pandemic viruses progressively evolve into seasonal viruses that acquire mutations to escape the immune response elicited in the previous year [76]. These antigenic changes require an annual update of the seasonal vaccine composition [77]. Interestingly, every 38–40 years, a replacement of the normally circulating seasonal virus with a completely new virus occurs that is not recognized by memory B and T lymphocytes and, thus, causes a pandemic, as most of the population is immunologically naïve [78].

Influenza virus infections induce both innate and adaptive host immune responses, which ultimately result in the abortion of virus replication [79]. Innate immunity and adaptive immunity profoundly differ from each other in terms of responsiveness, specificity and functionality. Innate immunity is the first line of defense against IAV that is specialized in controlling primary infection and induces the adaptive response through the production of co-stimulatory molecules, such as type I IFN, that exhibit antiviral, anti-proliferative and immunomodulatory functions [80]. Thus, antibody-mediated immunity and cellular-mediated immunity become activated and completely neutralize the virus.

### 4.2. Mechanism of IAV Restriction by TRIM22

IAV induces type I IFNs and ISGs with an antiviral function [81,82,83]. Among these, TRIM22 restricts seasonal IAVs by interacting with the viral NP. The viral NP is a major structural component of the viral ribonucleoprotein (vRNP), a heterotrimeric complex that is bound to the viral RNA and is responsible for viral transcription and replication [84]. In particular, NP binding to viral RNA is crucial for vRNP activity during the elongation phase of vRNA transcription [85]. NP is required to stabilize nascent RNA, which would otherwise be degraded by host cell nucleases. TRIM22 binding to NP promotes its downregulation through ubiquitination and degradation in a proteosome-dependent manner [86]. The TRIM22 RING domain with its E3 ubiquitin ligase activity catalyzes the ligation of previously activated ubiquitin to the lysine residues of the NP [87].

### 4.3. TRIM22 and IAV Evolution

**Figure 2:**
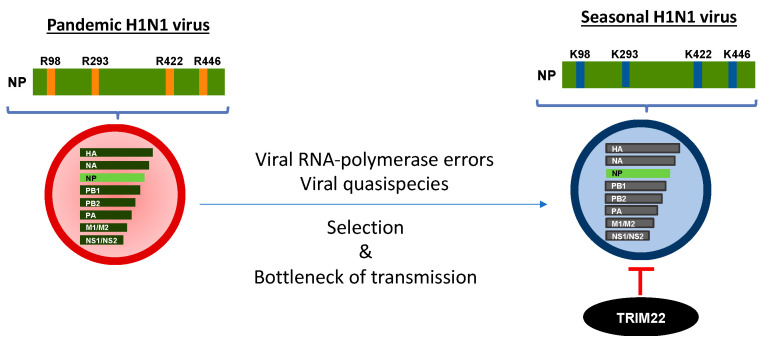
Evolution from pandemic to seasonal IAV has shaped TRIM22 restriction. Pandemic viruses are resistant to TRIM22 inhibition as their NP is endowed with four arginine (R) residues that progressively mutate into lysine (K) residues, becoming the target of the U3 ubiquitin ligase activity of TRIM22. The transition of R into K is dependent on viral polymerase errors that generate viral quasispecies either characterized by one, two, three or four K residues. However, a bottleneck of transmission favors the emergence of an IAV NP susceptible to TRIM22 restriction. This phenomenon is likely related to the general rule of viral evolution which endows the virus with the ability to become more transmissible and less pathogenic.

A wide range of proteomic and genome-wide RNAi-based screens have been used to identify host factors that are partners of NPs and RNPs in viral replication, as reviewed in [88]. However, few factors have been extensively characterized. TRIM22 has the peculiarity of being able to restrict seasonal, but not pandemic, influenza virus replication in vitro [89]. Despite the fact that the NP is a highly conserved protein, differently from the hemagglutinin protein that mediates entry into cells, and that it is the target of neutralizing antibodies [90], in comparison with seasonal pandemic virus sequences, four lysine (K) mutations were identified in seasonal viruses, whereas pandemic viruses were endowed with arginine (R) residues (Figure 2).

These four R-to-K changes progressively accumulated in approximately 90 years of IAV circulation in humans when sequences from the original pandemic 1918 H1N1 virus were compared with those of the following seasonal strains until 2009, when a new pandemic H1N1 virus emerged. The modeling of the atomic NP 3D structure showed that the four lysine residues are exposed to the solvent and therefore are potential targets of TRIM22 ubiquitination [89]. Concerning the other possible roles of the amino acid R-to-K changes, it has been previously reported that none of these residues are involved in the bipartite nuclear localization signal [91], binding to viral RNA [92,93] and viral polymerases [94], but they are mainly correlated with the host specificity of the virus [95]. In this regard, two sites, i.e., 98 and 422, are part of cytotoxic T lymphocyte (CTL) epitopes [96]. As only two of the four R-to-K variations are likely the result of CTL escape, other selective forces must contribute to the NP variation.

Of relevance is the potential role of adaptive mutations in the IAV animal host that can render viruses resistant to human restriction factors and, thus, have the advantage of being transmitted to humans. In this regard, human myxovirus resistance A (MxA) has been described as a potent restriction factor of avian IAVs [97]; however, the 1918 and 2009 pandemic H1N1 viruses have acquired a cluster of mutations in the NP that inactivates MxA restriction [98]. Mutations conferring MxA resistance are absent in avian IAVs; however, these mutations have been acquired in avian-derived viruses circulating in swine [99]. As pandemic strains are also resistant to TRIM22 restriction, NP adaptation in the swine host could also explain their lack of susceptibility to TRIM22 restriction. However, during IAV evolution in humans, TRIM22 acquires the ability to interact with the NP and adaptive mutations in the NP that render IAVs sensitive to TRIM22 restriction. Indeed, TRIM22 directly interacted with the NP of susceptible IAV strains both in a cotransfection system and during infection, and this interaction was followed by TRIM22-mediated downregulation and ubiquitination of the viral protein [86]. In contrast, the 2009 pandemic virus and the viral strains that are resistant to TRIM22 activity were unable to interact with TRIM22. Experiments based on the mini-replicon genome system demonstrated that the four NP R-to-K mutations are the main determinants of TRIM22 sensitivity [89].

In order to elucidate the mechanisms that IAV has adopted to escape restriction factors, Juan Ortin’s laboratory demonstrated that, in the absence of the selection pressure exerted by IFNs, serial passages of IAV promoted the introduction of mutations that allowed the virus to increase replication fitness [100]. However, in the absence of any constraint such as that of IAV cultivation in eggs or cell cultures, many of the adaptative mutations acquired during viral passages were purged from the viral population during or shortly after infection, as demonstrated in a human challenge study [101]. In the presence of selection pressure and the bottleneck of transmission, IAV may acquire adaptative mutations that could lead to increased susceptibility to restriction factors, including TRIM22, thereby resulting in a less efficient viral replication.

In conclusion, TRIM22 is an IFN-dependent restriction factor of human-adapted IAV, whereas it does not function as a barrier for pandemic viral strains. During replication in animal hosts, the pandemic strains undergo a number of amino acid changes in the NP that render them resistant to TRIM22 restriction and favor their transmission and human-to-human spread. Overall, the genetic variations in the NP gene will be useful for monitoring the viruses and preparing effective prevention and control strategies for potential pandemic influenza outbreaks.

## 5. Other RNA and DNA Viruses

TRIM22 restricts multiple RNA viruses (Table 1), including encephalomyocarditis virus (EMCV) [87] and hepatitis C virus (HCV) [102]. Early studies from Eldin and colleagues identified TRIM22 as a potent inhibitor of EMCV replication (Table 1) [87]. EMCV is a small non-enveloped RNA virus that belongs to the family of *picornaviridae*. EMCV infects a myriad of animals including wild and domestic animals [103]. Indeed, EMCV has often been described as a potential zoonotic agent, and although infection in humans is not frequent, it might be more common than expected as most human cases are asymptomatic and/or unrecognized [104]. TRIM22 was shown to interact directly with the EMCV 3C protease (3C^PRO^), a fundamental protein involved in the processing of both capsid and non-structural proteins, and in the inhibition of host immune responses. This interaction allows the polyubiquitination of 3C^PRO^ by TRIM22 that is delivered to the proteasome machinery, reducing EMCV virus replication [87].

HCV is single-stranded RNA of the *flaviviridae* family that causes persistent infection, leading to liver cirrhosis or hepatocellular carcinoma [105]. Treatment of HCV-infected individuals with IFN-α induced high levels of TRIM22 expression in PBMC [106]. In vitro studies have shown that TRIM22 binds to NS5A, a viral protein that counteracts the IFN-α signaling pathway induced by the infection [107]. This interaction results in NS5A downregulation mediated by the polyubiquitination of NS5A, and its degradation by the proteasome [102].

Very recently, a report has shown that TRIM22 exerts an inhibitory activity on respiratory syncytial virus (RSV) [108]. RSV is a negative single-stranded RNA virus that belongs to the family of *paramyxoviridae*. RSV circulates during the winter season and causes severe respiratory tract illness in infants, remaining a significant cause of death [109]. The evidence of TRIM22 antiviral activity against RSV is founded on in vitro infection of permissive cells in which TRIM22 expression was downregulated by RNA interference. An increase in viral RNA expression was detected in TRIM22 knocked-down cells, as compared to control cells [108]. However, the mechanism of RSV restriction by TRIM22 remains to be elucidated.

Furthermore, TRIM22 expression is modulated in response to several other viruses and viral antigens. TRIM22 is upregulated during the clearance of HCV in chimpanzees [110], and in response to infection by rubella virus [111], but also DNA viruses such as Epstein–Barr virus (EBV) [112], although it was downregulated during infection with another DNA virus, human papillomavirus type 31 [28].

Overall, these findings support the hypothesis that TRIM22 is involved in the host antiviral response, and the list of restricted viruses might increase in the future.

**Table 1:** TRIM22 restriction of RNA and DNA viruses.

Family	Virus	Genome	Mechanism of Restriction	Ref.
*Retroviridae*	HIV-1	ssRNA (+)	Gag trafficking, transcriptional silencing	[63,66]
*Ortomyxoviridae*	IAV	ssRNA (−)	Ubiquitination of NP	[86,89]
*Picornaviridae*	EMCV	ssRNA (+)	Ubiquitination of 3C protease	[87]
*Flaviviridae*	HCV	ssRNA (+)	Ubiquitination of NS5A	[102]
*Pneumoviridae*	RSV	ssRNA (−)	ND	[113]
*Hepadnaviridae*	HBV	dsDNA	Transcriptional repression	[31]
*Herpesviridae*	HSV-1	dsDNA	Epigenetic silencing	[114]
*Herpesviridae*	EBV	dsDNA	LMP1 induces TRIM22	[112,114]
*Herpesviridae*	KSHV	dsDNA	LANA induces TRIM22	[115]

ND means not determined.

TRIM22 is also characterized by its ability to inhibit HBV gene expression and replication, both in hepatocellular carcinoma cultured cells (HepG2 cells), and in mice (Table 1). HBV is a partially double-stranded DNA virus that belongs to the family of *hepednaviridae.* HBV causes hepatitis and liver cirrhosis [116] and remains the most important global risk factor of hepatocellular carcinoma [117]. TRIM22 inhibited the activity of the HBV core promoter (CP), which plays a central role in HBV replication. Furthermore, its antiviral activity resides on its B30.2 domain-mediated nuclear localization, and on the RING domain activity [31], although this finding has not been confirmed by a study demonstrating the anti-HBV activity of another family member, TRIM41 [118]. Nevertheless, more recent studies have been focused on the mechanisms of viral evasion from TRIM22. The HBV X (Hbx) protein is a viral factor with a multitude of effects on hepatocyte physiology, which range from interfering with cellular and HBV gene expression, and can modulate several signal transduction pathways [119,120]. Hbx was shown to inhibit TRIM22 transcription by promoting the methylation of a single CpG positioned in the TRIM22 5′ untranslated region, with a further reduction in the IRF-1 binding affinity [29].

Herpesviruses are large-genome DNA viruses that are divided into three groups named alpha-, beta- and gamma-herpesviruses and include members that are the target of TRIM22 restriction. All three subfamilies encompass viruses that are pathogenic for humans, and that can also cause severe disease [121]. Among the alpha-herpesviruses, it has recently been demonstrated that herpes simplex virus I (HSV-1) is inhibited by TRIM22 [114]. The mechanism of inhibition relies on TRIM22 activity to silence viral DNA encoding immediate-early viral genes by promoting chromatin compaction. The deletion of the B30.2 domain abrogated TRIM22-mediated inhibition of HSV-1 replication similarly to TRIM22-mediated inhibition of HBV [31]. Among the gamma-herpesviruses, EBV was also shown to be inhibited by TRIM22. Previous studies have provided evidence that the latent membrane protein 1 (LMP1) of EBV induces an antiviral state by upregulating ISGs including TRIM22 [112]. More recently, TRIM22 was shown to reduce the efficiency of EBV infection [114]. Among gamma-herpesviruses, Kaposi sarcoma-associated herpesvirus (KSHV), also known as HHV8, has also been shown to induce TRIM22. KSHV causes a vascular tumor predominantly found in immunosuppressed individuals such as during HIV-1 infection [122]. In particular, the latency-associated nuclear antigen (LANA) has been shown to activate several ISGs including TRIM22 [115].

## 6. Discussion

Viral invasion in target cells is hindered by several host factors that are induced after virus sensing by the PPR. In particular, type I IFNs are responsible for inducing hundreds of genes with an antiviral function. TRIM22 is one of these genes that contributes to limiting viral infection. As in other tissues, TRIM22 is constitutively expressed in epithelial cells, but it is promptly induced by viral infection [123]. Consistent with the innate immunity effector intracellular proteins, TRIM22 is not specific to a single virus, but many DNA and RNA viruses are restricted, and, likely, many other still unknown viruses are sensitive to its antiviral action.

The mechanisms of TRIM22 antiviral restriction may depend on the RING domain that has E3 ubiquitin ligase activity, as demonstrated for IAV [86], HBV [31] and EMCV [87]. However, other mechanisms that involve the CC domain or the B30.2 domain, as for HIV-1 [20] and HSV-1, participate in its antiviral activity [114].

For the TRIM22 antiviral function, cellular localization likely plays an important role as TRIM22 restricts viruses that exploit both the cytoplasm and the nucleus for their replication. TRIM22 is active against viruses that replicate in the nucleus such as IAV, HBV and herpesviruses; however, TRIM22 also inhibits EMCV that replicates in the cytoplasm. This is consistent with the activity of other restriction factors that localize to and inhibit viruses in specific cellular compartments [37]. Of note is the high ratio of non-synonymous to synonymous nucleotide substitutions of TRIM22 that reflects signatures of positive selection [21]. Indeed, many restriction factors have evolved fast and show evolutionary signatures of adaptation to pathogenic viruses [124,125]. Regarding TRIM22, two SNPs have been correlated with in vitro HIV-1 infection and the severity of the disease [67]. Furthermore, signatures of viral evolution have been demonstrated in the IAV NP that undergoes nucleotide changes from pandemic to seasonal strains that favor NP ubiquitination and degradation, and, ultimately, TRIM22 restriction [89], contributing to the attenuation of viral pathogenicity typical of pandemic-to-seasonal virus transitions.

Given its constitutive expression in epithelial cells [123], of note is its potential role in the restriction of the novel SARS-CoV-2 that is causing the current paramount COVID-19 pandemic. In this regard, a recent study demonstrated the upregulation of TRIM22 expression in lung epithelial cells infected with SARS-CoV-2 in vitro [113]. Further experiments will be required to determine whether TRIM22 exerts restriction of SARS-CoV-2 replication.

In conclusion, TRIM22 acts as restriction factor of many viruses. The mechanism of its antiviral activity varies depending on the viral target, and almost any domain of the protein can execute antiviral activity. Cellular restriction factors are often counteracted by specific viral proteins [126,127]; however, with the exception of the Hbx protein of HBV [29], whether other viral antagonists of TRIM22 exist remains largely unknown.

## Data Availability

Not applicable.
